# Diffuse large B-cell lymphoma combined with two solid tumors: a case report

**DOI:** 10.3389/fonc.2025.1561923

**Published:** 2025-06-17

**Authors:** Xiufang Zhang, Shaojun Zhou, Yuqiao Zhou, Huiqing Qiu

**Affiliations:** ^1^ Department of Oncology, The Second People’s Hospital of Quzhou, Quzhou, China; ^2^ Department of Rehabilitative, The Second People’s Hospital of Quzhou, Quzhou, China

**Keywords:** multiple primary malignant tumors, DLBCL, prostate cancer, rectal cancer, solid tumors

## Abstract

We report a rare case of multiple primary tumors in a 67-year-old male. The patient was treated surgically for a lumbar disc herniation with intraoperative findings of L5 nerve root tumor, and postoperative pathology confirmed a diagnosis of diffuse large B-cell lymphoma (DLBCL). Three months later, the patient was further pathologically diagnosed with prostate cancer. The multidisciplinary treatment team devised a staged therapeutic strategy: firstly, 2 cycles of R-CHOP chemotherapy for DLBCL, followed by local radiotherapy for the residual lesion of lumbar spine tumor, and then 2 cycles of R-MTX-CHOP chemotherapy in sequence; after the DLBCL was controlled, the patient underwent radical prostatectomy, which was followed by adjuvant endocrine therapy and pelvic lymph node radiotherapy. Post-treatment evaluation showed complete remission (CR) in both tumors. After five years of disease-free survival, the patient presented with enlarged cervical lymph nodes in October 2022. A biopsy confirmed DLBCL recurrence. The treatment team administered six cycles of R-GemOx chemotherapy, followed by maintenance therapy with the targeted agent Orelabrutinib. Subsequent assessments confirmed a second CR. In September 2024, a colonoscopy revealed a rectal tumor, which was pathologically diagnosed as rectal cancer. The patient underwent radical surgery, and the pathological staging was determined to be stage I. According to the literature search, this is the first reported case of triple primary malignancies involving DLBCL, prostate cancer and rectal cancer, which provides valuable experience in the clinical diagnosis and treatment of such rare cases.

## 1 Introduction

Multiple Primary Malignant Tumors (MPMTs) refer to the occurrence of two or more histologically distinct malignant tumors in the same individual, with no evidence of recurrence, metastasis, or local spread between these tumors. Based on the time interval between the diagnosis of the second primary tumor and the first, MPMTs can be classified into simultaneous multiple primary malignancies (SMPMTs) and metachronous multiple primary malignancies (MMPMTs) (1). SMPMTs were defined as second primary tumors diagnosed within 6 months of the diagnosis of the first primary tumor, while MMPMTs were defined as second primary tumors diagnosed more than 6 months after the diagnosis of the first primary tumor (2). This case report describes a rare patient with both SMPMTs and MMPMTs. The patient was initially diagnosed with both DLBCL and prostate cancer and achieved long-term remission through standardized treatment. Approximately seven years later, the patient was diagnosed with rectal cancer, which met the diagnostic criteria for MMPMTs. This case not only exemplifies the complexity of MPMTs but also provides valuable insights into the clinical management of such rare conditions.

## 2 Case presentation

### 2.1 Initial visit and diagnosis

A 67-year-old male patient was admitted to the People’s Liberation Army No. 117 Hospital (Hangzhou, China) on December 6, 2016, presenting with “low back pain accompanied by tingling and numbness in the left lower limb persisting for over six months.” The patient had previously been in good health, with an Eastern Cooperative Oncology Group (ECOG) performance status score of 1 and a Numeric Rating Scale (NRS) pain score of 3. He denied systemic symptoms such as weight loss, night sweats, or persistent fever. There were no reported comorbidities, genetic or environmental exposure risk factors, or family history of cancer. Physical examination revealed significant limitation of lumbar movement, tenderness over the L3-L5 spinous processes and paravertebral regions (+), pain on percussion (+), radiating to the left lower limb, and a positive straight leg raising test on the left side (60°). Laboratory tests showed a normal white blood cell count and lymphocyte percentage, with negative results for human immunodeficiency virus (HIV) and Epstein-Barr virus DNA (EBV-DNA) testing. Magnetic resonance imaging (MRI) of the lumbar spine indicated lumbar disc herniation. The initial diagnosis was lumbar degenerative disease accompanied by disc herniation.

### 2.2 Initial surgery and pathological diagnosis

**Figure 1:**
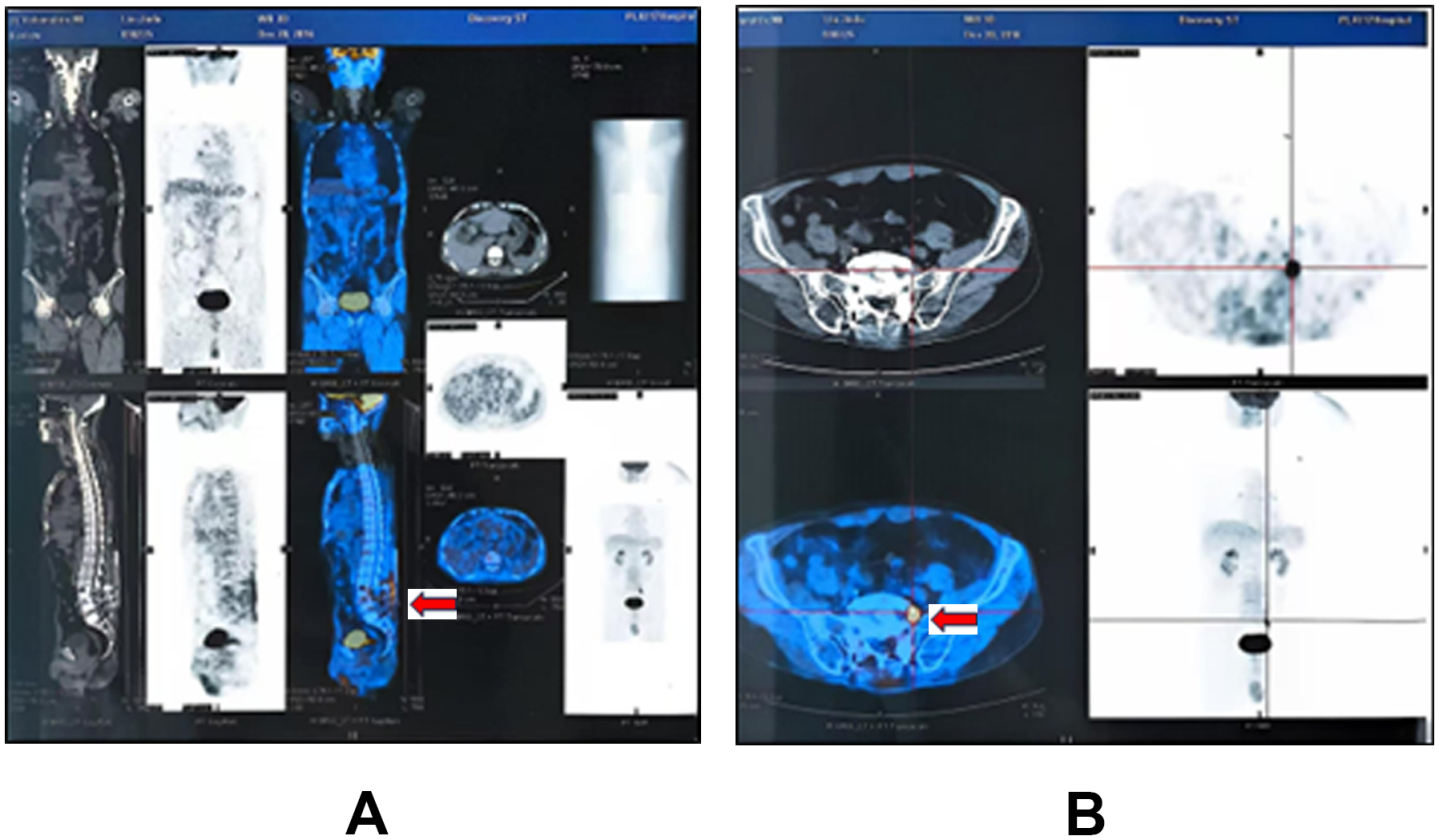
Postoperative PET-CT (December 2016): **(A)** Sagittal view showing patchy areas of moderately increased radiotracer uptake, with a maximum standardized uptake value (SUVmax) of 4.46 within the soft tissues and bone at the surgical site. **(B)** Axial view at the L5 level revealing thickening of the left L5 nerve root, more pronounced at the intervertebral foramen exit, with a SUVmax of 12.92.

On December 9, 2016, the patient underwent lumbar decompression and interbody fusion with internal fixation. During the procedure, a tumor was unexpectedly discovered at the L5 nerve root, leading to additional resection of the L5 nerve root tumor. Morphological and immunohistochemical analysis suggested diffuse large B-cell lymphoma (DLBCL), non-germinal center type. The specific immunophenotypic markers included: CD45R0 (**−**), CD79a (+), CKpan (**−**), EMA (**−**), LCA (+), Vimentin (+), Bcl-2 (+), Bcl-6 (**−**), CD10 (**−**), CD20 (+), Ki67 (+, 75%), and MUM1 (+). Fluorescence *in situ* hybridization (FISH) testing was negative for BCL-6, BCL-2, and C-MYC gene rearrangements. Postoperative PET-CT (December 20, 2016) revealed thickening of the left L5 nerve root with a segmental abnormal increase in FDG uptake, indicating residual tumor. Additionally, the prostate appeared abnormally enlarged, necessitating further investigation (**Figures 1A, B**). The final diagnosis was DLBCL (stage IE, non-germinal center type, IPI score 3, intermediate-high risk).

### 2.3 Diagnosis of prostate cancer

In March 2017, the patient visited the Second People’s Hospital of Quzhou (Quzhou, China) for prostate-related examinations. Results showed a total prostate-specific antigen (tPSA) level of 12.82 ng/mL, free prostate-specific antigen (fPSA) level of 1.95 ng/mL, and a free-to-total PSA ratio of 0.15. Prostate ultrasound revealed a space-occupying lesion. Prostate needle biopsy confirmed prostate adenocarcinoma with a Gleason score of 3 + 3 = 6. At this time, the patient was diagnosed with SMPMTs: DLBCL and prostate cancer.

### 2.4 Treatment and remission of DLBCL

On April 7, 2017, after a multidisciplinary team (MDT) discussion, priority was given to treating the more aggressive DLBCL. The patient received 2 cycles of R-CHOP chemotherapy, specifically: rituximab (375mg/m², d0), doxorubicin (50mg/m², d1), vincristine (1.4 mg/m², d1), cyclophosphamide (750 mg/m², d1), and prednisolone (100 mg, d1-5). Subsequently, intensity-modulated radiation therapy (IMRT) (30 Gy/15 F) was administered to the residual lumbar lesion. To consolidate efficacy, the patient received 2 additional cycles of R-MTX-CHOP chemotherapy, specifically: rituximab (375 mg/m², d0), methotrexate (3 g/m², d1), doxorubicin (50 mg/m², d1), vincristine (1.4 mg/m², d1), cyclophosphamide (750 mg/m², d1),and prednisolone (100 mg, d1-5), incorporating high-dose methotrexate. On November 7, 2017, follow-up PET-CT showed complete disappearance of the lumbar residual lesion, indicating complete remission (CR) of DLBCL.

### 2.5 Treatment and remission of prostate cancer

**Figure 2:**
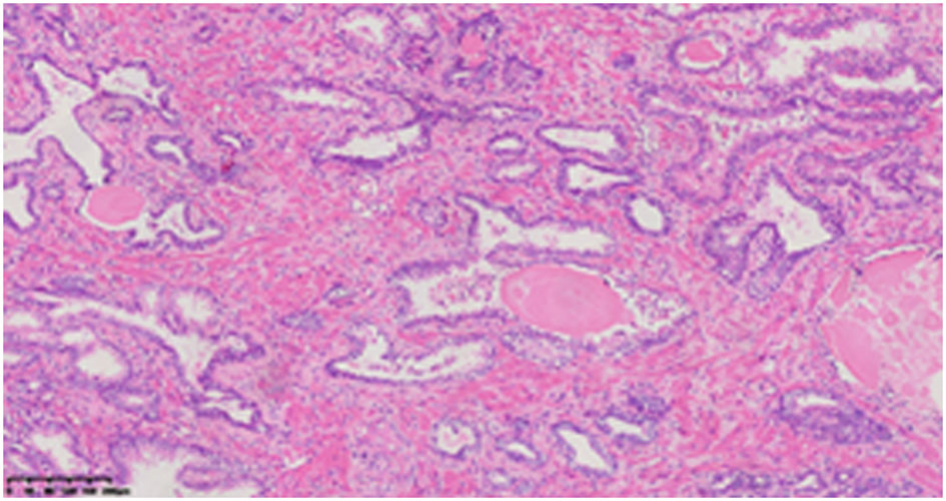
Histopathological examination (HE staining, ×10 magnification): Prostatic Adenocarcinoma (Acinar Type), Gleason Score 3 + 4 = 7: The tumor involves both lobes of the prostate, with perineural invasion and tumor infiltration close to the peripheral surgical margin.

On December 1, 2017, the patient underwent laparoscopic radical prostatectomy. Postoperative pathology revealed a Gleason score of 3 + 4 = 7, Grade Group 2, with a pathological stage of T3aN0M0, stage IIIB (**Figure 2**). Postoperatively, the patient received bicalutamide 50 mg orally once daily and triptorelin acetate 3.75 mg intramuscularly for endocrine therapy over 18 months, along with IMRT (55 Gy/25 F) to the pelvic lymph drainage area. On October 23, 2018, follow-up PET-CT confirmed CR of both prostate cancer and DLBCL, marking the transition to regular follow-up.

### 2.6 DLBCL recurrence and treatment

**Figure 3:**
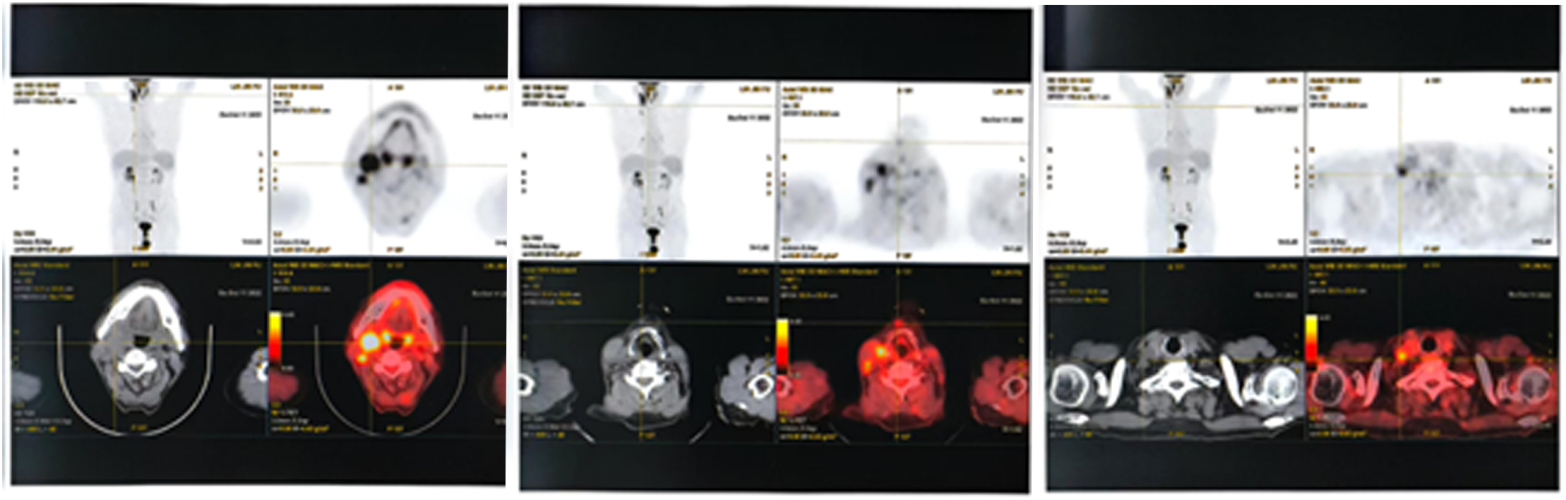
PET-CT Findings (October 2022): Multiple enlarged lymph nodes were observed in the right submandibular region, along the medial border of the sternocleidomastoid muscle in the deep cervical group, and in the right supraclavicular fossa. The largest node measured approximately 2.7 cm in short-axis diameter, with an average SUVmax of 6.5 and a maximum SUVmax of 7.5. These findings are suggestive of lymphoma infiltration.

**Figure 4:**
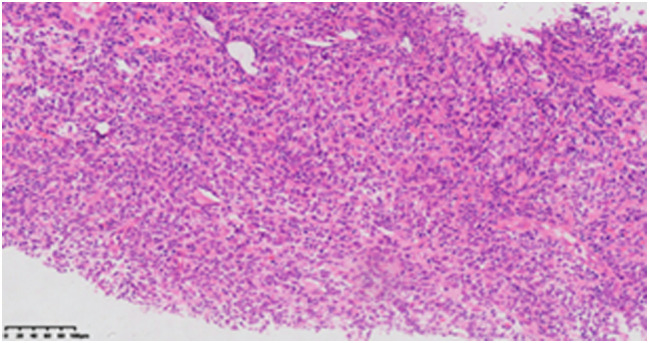
Cervical lymph node biopsy histopathological examination (HE staining, ×10 magnification) reveals diffuse abnormal hyperplastic lesions of lymphoid tissue, suggestive of lymphoma.

In October 2022 (5 years later), routine follow-up revealed multiple enlarged lymph nodes in the right submandibular, deep cervical group along the medial border of the sternocleidomastoid muscle, and right supraclavicular fossa. PET-CT showed significantly increased FDG uptake, suggesting lymphoma infiltration (**Figure 3**). A needle biopsy of the cervical lymph nodes (**Figure 4**) with immunohistochemistry results included: CK (-), CD3 scattered (+), CD20 (+), CD21 (**−**), Bcl-2 (+), CyclinD1 (**−**), CD56 (**−**), CD138 scattered (+), CD10 (**−**), MUM1 scattered (+), EBER (**−**), and Ki-67 (+, 60%). These findings confirmed DLBCL recurrence. The patient subsequently received 6 cycles of R-GemOx chemotherapy, specifically: rituximab (375 mg/m², d0), gemitabine (1000 mg/m², d1), and oxaliplatin (100 mg/m², d1). After chemotherapy, orelabrutinib was administered as targeted maintenance therapy.

### 2.7 Rectal cancer and treatment

**Figure 5:**
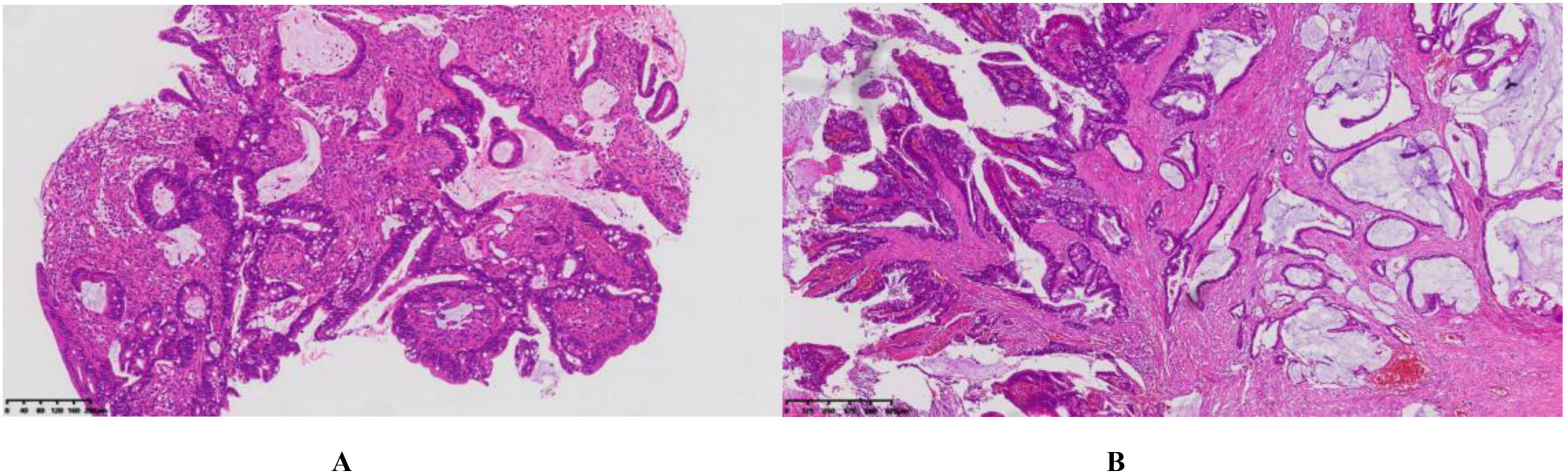
**(A)** Colonoscopy and histopathological examination (HE staining, ×10 magnification) **(B)** Postoperative pathological examination (HE staining, ×4 magnification): Moderately differentiated adenocarcinoma with partial mucinous adenocarcinoma. The tumor measures approximately 1.5 cm × 1 cm × 0.5 cm, infiltrates the deep muscular layer, and shows no clear evidence of vascular or perineural invasion.

During routine follow-up in September 2024, colonoscopy revealed a rectal lesion, and pathological examination confirmed adenocarcinoma (**Figure 5A**). The patient subsequently underwent laparoscopic radical rectal surgery. Postoperative pathology indicated moderately differentiated adenocarcinoma, with a pathological stage of pT2N0M0, Stage I (**Figure 5B**).

In October 2024, the PET-CT showed no signs of tumor recurrence or metastasis. The treatment efficacy was assessed as CR. Currently, the patient continues maintenance therapy with orelabrutinib. The patient is in good general condition, with an ECOG performance status score of 1, and maintains a good quality of life.

## 3 Discussion

The incidence of MPMTs has significantly increased with advancements in cancer diagnosis and treatment, as well as prolonged patient survival. The reported incidence of MPMTs ranges from 0.73% to 11.7% (3). The treatment principles for MPMTs require individualized planning based on the stage, pathological type of each tumor, and the patient’s overall condition. When pathologically confirmed, each tumor should be evaluated and staged independently. Given the potential for confusion between MPMTs and metastatic cancer, multidisciplinary team (MDT) discussions are crucial for challenging cases. Generally, tumors with a greater impact on patient survival or quality of life should be prioritized. We present a representative case of MPMTs, involving the concurrent diagnoses of DLBCL and prostate cancer, followed by a diagnosis of rectal cancer 7 years later. In this case, PET-CT and pathological biopsies were used to establish the diagnosis, and an individualized treatment plan was tailored according to the tumor type and stage.

### 3.1 Analysis of treatment strategies

The R-CHOP regimen, as a first-line chemotherapy for DLBCL, achieved significant efficacy in this case. Combined with local radiotherapy, the patient achieved a 5-year disease-free remission. Upon relapse, the R-GemOx regimen was employed as second-line therapy, followed by orelabrutinib maintenance treatment.

Orelabrutinib, a second-generation BTK inhibitor, has shown potential clinical efficacy in chronic lymphocytic leukemia (CLL), small lymphocytic lymphoma (SLL), mantle cell lymphoma (MCL), and relapsed or refractory Waldenström macroglobulinemia (4–6). However, its role in DLBCL remains exploratory. Retrospective studies suggest high response rates with orelabrutinib combined with chemotherapy in both untreated and relapsed patients (7, 8). To date, no data exists on orelabrutinib maintenance therapy in DLBCL. The successful treatment of this patient provides valuable insights into its potential application in DLBCL maintenance.

### 3.2 Analysis of potential factors in MPMTs

#### 3.2.1 Genetic factors

Genetic factors play a central role in MPMTs. Known hereditary cancer syndromes, such as Lynch Syndrome, BRCA1/2 mutations, and TP53 mutations, are closely associated with the development of multiple malignancies (9–11). Lynch Syndrome, which is caused by mutations in DNA mismatch repair genes (e.g., MLH1, MSH2, MSH6, PMS2), significantly increases the risk of colorectal, endometrial, and other cancers. Similarly, BRCA1/2 mutations are linked not only to breast and ovarian cancers but also to prostate and pancreatic cancers (11–13). Although comprehensive genetic testing was not performed in this case, the sequential diagnoses of DLBCL, prostate cancer, and rectal cancer suggest the possibility of an unrecognized hereditary cancer syndrome. Therefore, comprehensive genetic testing and family history investigation are recommended for patients with MPMTs to identify potential hereditary etiologies.

#### 3.2.2 Immune system and tumor microenvironment

The immune system plays a critical role in cancer development and progression. Chronic inflammation and immunosuppression may promote tumorigenesis, while abnormal immune activation can impair immune surveillance, increasing cancer risk (14). Immune cells in the tumor microenvironment play a key role in cancer development and progression (15). For instance, M1/M2 macrophage polarization and tumor-associated macrophages (TAMs) promote tumor growth through cell proliferation, immune suppression, and angiogenesis (16). The initial diagnosis of DLBCL in this patient indicates B-cell dysfunction, which may have contributed to the development of other tumors due to immune dysregulation. Therefore, immune system assessment and modulation are essential in MPMTs management.

#### 3.2.3 Treatment-related factors

Radiotherapy and chemotherapy, as primary cancer treatments, increase the risk of secondary primary malignancies through various mechanisms due to their long-term side effects (17, 18). Studies show a significantly elevated risk of rectal cancer in patients who received radiotherapy (OR: 1.45, 95% CI: 1.07-1.97, P = 0.02) (19), consistent with this case. Certain chemotherapeutic agents (e.g., alkylating agents, anthracyclines) may also increase secondary tumor risk through DNA damage or immune suppression (20). Thus, treatment plans should balance therapeutic benefits with long-term risks to minimize MPMTs occurrence.

#### 3.2.4 Environmental and lifestyle factors

Chronic exposure to carcinogens (e.g., tobacco, alcohol, chemical pollutants) and unhealthy lifestyle habits (e.g., high-fat diet, physical inactivity) may increase the risk of multiple cancers (21, 22). For example, smoking is associated with lung cancer and may also elevate the risk of bladder and pancreatic cancers (23). For MPMTs patients, a detailed investigation of lifestyle and occupational exposures is necessary.

## 4 Conclusion

We report a case of MPMTs involving concurrent DLBCL and prostate cancer, with a subsequent diagnosis of rectal cancer 7 years later. The patient achieved complete remission at all stages following systematic treatment. The successful use of orelabrutinib in DLBCL maintenance therapy provides valuable insights for future research. The occurrence of MPMTs may be related to genetic factors, immune dysfunction, long-term side effects of radiotherapy and chemotherapy, as well as environmental and lifestyle factors. For patients with MPMTs, comprehensive genetic testing and family history investigation are crucial, and optimizing treatment strategies is essential for reducing the risk of secondary tumors. Furthermore, enhanced long-term follow-up and preventive measures are essential.

## Data Availability

The original contributions presented in the study are included in the article/Supplementary Material. Further inquiries can be directed to the corresponding authors.
